# Inflammation Related to Association of Low Uric Acid and Progression to Severe Disease in Patients Hospitalized for Non-Severe Coronavirus Disease 2019

**DOI:** 10.3390/biomedicines11030854

**Published:** 2023-03-10

**Authors:** Masafumi Kurajoh, Yoshikazu Hiura, Ryutaro Numaguchi, Yasutaka Ihara, Takumi Imai, Tomoaki Morioka, Masanori Emoto, Yukio Nishiguchi

**Affiliations:** 1 Department of Metabolism, Endocrinology and Molecular Medicine, Osaka Metropolitan University Graduate School of Medicine, Osaka 545-8585, Japan; 2 Department of Diabetes and Endocrinology, Osaka City Juso Hospital, Osaka 532-0034, Japan; 3 Department of Medical Statistics, Osaka Metropolitan University Graduate School of Medicine, Osaka 545-8585, Japan; 4 Department of Surgery, Osaka City Juso Hospital, Osaka 532-0034, Japan; 5 Directors Office, Osaka City General Hospital, Osaka 534-0021, Japan

**Keywords:** uric acid, antioxidants, inflammation, COVID-19, disease progression

## Abstract

Uric acid has antioxidant properties. To examine whether a low uric acid level is associated with severe coronavirus disease 2019 (COVID-19) progression via inflammation, alveolar damage, and/or coagulation abnormality, a retrospective observational study of 488 patients with non-severe COVID-19 and serum uric acid level ≤7 mg/dL at admission was conducted. Serum C-reactive protein (CRP), serum Krebs von den Lungen 6 (KL-6), and plasma D-dimer levels were also measured as markers of inflammation, alveolar damage, and coagulation abnormality, respectively. Median values for uric acid, CRP, KL-6, and D-dimer at admission were 4.4 mg/dL, 3.33 mg/dL, 252.0 U/mL, and 0.8 µg/mL, respectively. Among the total cohort, 95 (19.5%) progressed to severe COVID-19 with a median (interquartile range) time of 7 (4–14) days. Multivariable Cox proportional hazards regression analysis showed that low uric acid level was associated with a higher rate of severe COVID-19 progression. However, uric acid level was inversely associated with CRP level, and the association between the level of uric acid and severe COVID-19 progression was significantly different with and without CRP level inclusion. In contrast, no such association was found for KL-6 or D-dimer level. Low uric acid may contribute to severe COVID-19 progression via increased inflammation in subjects without hyperuricemia.

## 1. Introduction

Worldwide, the numbers of coronavirus disease 2019 (COVID-19) cases caused by severe acute respiratory syndrome coronavirus 2 (SARS-CoV-2) and showing progression to increased severity have been reported to be increasing [1,2,3]. Accumulating evidence also indicates that pathophysiological factors considered to be related to oxidative stress [4,5], including inflammation, alveolar damage, and coagulation abnormality, underlie the development of severe COVID-19 cases [6,7].

Uric acid has been shown to have an ability to gain antioxidant properties by scavenging reactive oxygen species (ROS) as well as pro-oxidant properties by generating ROS [8,9,10,11]. Of interest, several studies have found that not only high but also low uric acid levels were associated with an increased incidence of severe COVID-19 progression, such as in cases with mechanical ventilation, intensive care unit (ICU) admission, and/or composite outcome, as well as COVID-19-related mortality such as in-hospital death [12,13,14], while a reduction in the antioxidant property is considered to be involved in those associations in patients with a low level of uric acid. The administration of uric acid precursors has been indicated to suppress inflammatory mediators thorough ROS elimination in cases of indomethacin-induced enteropathy [15]; thus, it is speculated that low uric acid may contribute to COVID-19 severity via inflammation. However, to the best of our knowledge, no study has been conducted to comprehensively examine whether the association of a low uric acid level with severe COVID-19 progression is related to inflammation, alveolar damage, and/or coagulation abnormality.

This study aimed to clarify the role of reduced uric acid in progression to severe COVID-19. To achieve this objective, we examined the records of patients initially hospitalized for non-severe COVID-19 and with a serum uric acid level ≤7 mg/dL at the time of admission. From the data obtained, associations of serum uric acid level with (1) severe COVID-19 incidence and (2) markers of inflammation, alveolar damage, and coagulation abnormality, as well as (3) the effects of markers of inflammation, alveolar damage, and coagulation abnormality on the association of serum uric acid level with severe COVID-19 progression were analyzed. Although there are various markers related to inflammation, alveolar damage, and coagulation abnormality, it has been reported that serum C-reactive protein (CRP), serum Krebs von den Lungen 6 (KL-6), and plasma D-dimer levels, respectively, are specific useful markers of those, being used similarly for COVID-19 cases [16,17,18]. Therefore, in the present study, serum CRP was used as a marker of inflammation, serum KL-6 as a marker of alveolar damage, and plasma D-dimer as a marker of coagulation abnormality.

## 2. Materials and Methods

### 2.1. Study Design

This observational retrospective study analyzed patients treated at Osaka City Juso Hospital, designated as a priority medical institution for COVID-19 by the Osaka Prefectural Government, and the first to focus on patients with non-severe COVID-19 in Japan. This investigation was conducted in full accordance with the principles of the Declaration of Helsinki and Ethical Guidelines for Clinical Studies by the Ministry of Health, Labor and Welfare, Japan. The protocol, which included anonymization of patient information, was approved by the Ethics Committee of Osaka City Juso Hospital on 23 June 2021 (No. 3-A1) and the Graduate School of Medicine of Osaka City University, which were merged in 2022 to form Osaka Metropolitan University on 5 October 2021 (approval No. 2021-159). 

### 2.2. Inclusion and Exclusion Criteria

Inclusion and exclusion criteria for the present study are discussed in the following section. Patients admitted to Osaka City Juso Hospital for non-severe COVID-19 between October 2020 and May 2021 were considered eligible for inclusion in this study. Exclusion criteria included those (1) with hyperuricemia, i.e., serum uric acid level at admission >7.0 mg/dL [19], (2) with severe COVID-19 at the time of admission, or (3) transferred to our hospital from a tertiary hospital following improvement from severe to non-severe COVID-19. Additionally, those who (4) received an immunosuppressive agent, (5) were pregnant, (6) self-discharged, or (7) were missing important data were not included. Since the purpose of this study was to analyze the significance of a low blood level of uric acid in cases of severe COVID-19 progression, treatment with a uric acid-lowering agent was not included in the exclusion criteria.

### 2.3. Determination of Uric Acid, CRP, KL-6, and D-Dimer Blood Levels

Blood parameters including uric acid, CRP, KL-6, and D-dimer levels were routinely measured using a sample obtained at the time of admission, with or without fasting [20,21]. Serum uric acid was measured by use of a uricase-N-(3-sulfopropyl)-3-methoxy-5-methylaniline assay (L-Type UA M; FUJIFILM Wako Pure Chemical Corporation, Osaka, Japan), serum CRP by use of a latex-enhanced immunoturbidimetric assay (IATRO CRP-EX; LSI Medience Corporation, Tokyo, Japan), and serum KL-6 by use of a latex immunoturbidimetric assay (Nanopia KL-6; Sekisui Medical Co. Ltd., Tokyo, Japan), with analyses of those performed with an automated biochemical analyzer (LABOSPECT 008; Hitachi High-Technologies, Tokyo, Japan). Plasma D-dimer was measured using a latex immunoturbidimetric assay (Nanopia D-dimer; Sekisui Medical Co. Ltd., Tokyo, Japan), with analysis performed with an automated coagulation analyzer (SYSMEX CS-2500; Sysmex Corporation, Kobe, Japan). The reference values for CRP, KL-6, and D-dimer levels were ≤0.3 mg/dL, <500 U/mL, and ≤1.0 µg/mL, respectively.

### 2.4. Diagnosis of COVID-19

The diagnosis of each patient was obtained using methods based on clinical practice guidelines for COVID-19 published by the Japanese Ministry of Health, Labour and Welfare [22,23]. Briefly, COVID-19 diagnosis was obtained utilizing nucleic acid amplification testing procedures, including a real-time polymerase chain reaction (PCR) assay, loop-mediated isothermal amplification, and transcription-mediated amplification, and also confirmed with quantitative or qualitative antigen test results for SARS-CoV-2, as approved for use at hospitals or clinics by the Japanese Ministry of Health, Labour and Welfare. 

### 2.5. Classification of COVID-19 Severity Level

Severity classification was determined according to oxygenation and respiratory symptoms. Patients with a percutaneous oxygen (SpO_2_) saturation ≥96%, and no respiratory symptoms, or coughing only without shortness of breath were classified as mild; those with an SpO_2_ saturation ranging from 93% to 96%, shortness of breath, and pneumonia findings were classified as moderate I (no respiratory failure); those with SpO_2_ saturation ≤93% and requiring O_2_ administration were classified as moderate II (respiratory failure); and those admitted to the ICU or who required a mechanical ventilator were classified as severe. Using those classifications, mild, moderate I, and moderate II cases were defined as non-severe COVID-19 for the present study [22,23]. 

### 2.6. COVID-19 Patient Management during Hospitalization

The present patients were treated according to guidelines current at that time [22,23]. For discharge from a bed for COVID-19, the following criteria were used: (i) 10 days from symptom onset date and 72 h after resolution of symptoms, or (ii) for patients with negative results of PCR or quantitative antigen testing performed twice with at least 24 h between the tests, 24 h following symptom resolution [22,23]. Patients who showed progression from non-severe to severe COVID-19 were transferred to a hospital classified as specialized for treatment and had an ICU bed available, except those who did not require life-prolonging therapy.

### 2.7. Outcome

The outcome for the present cases was based on period (days) between hospital admission and severe COVID-19 status progression. Any patient who did not progress to severe and met the discharge criteria was noted as not demonstrating progression to severe status following discharge, as previously described [21,24]. 

### 2.8. Other Clinical Assessments

Dates of onset of COVID-19 based on symptom appearance, use of medication, present and past illness, current smoking habit, height, and body weight for each subject were noted. Weight (kg) in kilograms divided by height in meters squared (kg/m^2^) was used to determine body mass index (BMI). An equation previously described for Japanese subjects was used to calculate estimated glomerular filtration rate (eGFR) [25]. Diagnosis of diabetes, hypertension, or dyslipidemia was performed according to history of treatment for the condition, or the American Diabetes Association, Japanese Society of Hypertension, or Japan Atherosclerosis Society guidelines [26,27,28]. 

### 2.9. Statistical Analysis

Due to the exploratory nature of the study, sample size calculation was not performed and all available data for the patients during the study period were used. Data representing clinical characteristics and baseline demographics are shown as median values (interquartile range [IQR]) for continuous variables, or number (percentage) for categorical variables. A multivariable Cox proportional hazards regression model was employed to examine the association of serum uric acid level with progression from non-severe to severe COVID-19, with adjustments made for potential confounding factors, noted as the following: age, sex, BMI, smoking habit, diabetes mellitus, hypertension, dyslipidemia, cerebrovascular and/or cardiovascular disease, chronic respiratory disease, eGFR, days from onset of disease to admission, COVID-19 severity at time of admission, and uric acid-lowering agent usage. Next, the consistency of the findings was analyzed after subgrouping the subjects based on sex (male, female) and uric acid-lowering agent usage (presence, absence). As for the associations of serum uric acid level with serum CRP, serum KL-6, and plasma D-dimer levels, analyses were performed using Pearson’s correlation coefficient and multivariable linear regression, with adjustments for the same factors as noted in the above-mentioned multivariable Cox proportional hazards regression model. Additionally, associations of serum CRP, serum KL-6, and plasma D-dimer level with progression to severe COVID-19 were examined using the same multivariable Cox proportional hazards regression model used for serum uric acid level. Finally, the relationship between uric acid level in serum and non-severe to severe COVID-19 progression was analyzed with an additional adjustment for serum CRP, serum KL-6, and plasma D-dimer level. Any change in serum uric acid hazard ratio (HR) indicating progression from non-severe to severe COVID-19, shown by these additional adjustments, was evaluated using a nonparametric bootstrap method with 1000 replications [29,30]. The values for serum CRP, KL-6, and plasma D-dimer levels were transformed logarithmically before the simple and multivariable regression analyses performed due to the skewed distribution.

For all data analyses, the R software package, version 3.6.3 (R Foundation for Statistical Computing, Vienna, Austria), was used. Presented *p* values are two-tailed, with <0.05 considered to indicate statistical significance.

## 3. Results

### 3.1. Study Population

During the study period, 573 patients with COVID-19 were admitted to Osaka City Juso Hospital. Patients with a serum uric acid level at admission >7.0 mg/dL were excluded (*n* = 33). In addition, those with severe COVID-19 at the time of admission (*n* = 5) or transferred to our hospital from a tertiary hospital following improvement from severe to non-severe COVID-19 (*n* = 28) were not analyzed for this study. Finally, patients who were receiving an immunosuppressive agent (*n* = 9), pregnant (*n* = 1), self-discharged (*n* = 1), or missing important data (*n* = 8) were not included in the analyses. Thus, a total of 488 patients (254 males, 234 females) with non-severe COVID-19 and a serum uric acid level ≤7 mg/dL at the time of admission were enrolled in this retrospective observational study as subjects.

### 3.2. Clinical Characteristics of Patients

The characteristics of the enrolled patients (*n* = 488) are presented in Table 1. The median value for uric acid for all was 4.4 mg/dL, and a uric acid-lowering agent was administered to 54 (11.1%). CRP, KL-6, and D-dimer median values were 3.33 mg/dL, 252.0 U/mL, and 0.8 µg/mL, respectively. The numbers of patients classified as mild, moderate I, and moderate II COVID-19 were 211 (43.2%), 167 (34.2%), and 110 (22.5%), respectively.

### 3.3. Medications Given for COVID-19 after Hospitalization

The medications given to the present COVID-19 patients included favipiravir in 370 (75.8%), steroids in 303 (62.1%), ciclesonide in 183 (38.4%), remdesivir in 24 (4.9%), and baricitinib in 2 (0.4%).

### 3.4. Progression from Non-Severe to Severe COVID-19

Ninety-five (19.5%) of the present 488 patients with non-severe COVID-19 at the time of hospitalization progressed to severe COVID-19, while 393 (80.5%) were discharged. From hospital admission to severe COVID-19 progression there was a median term of 7 days (IQR 4–14, range 1–40), while the median term to discharge was 12 days (IQR 9–16, range, 3–47). 

### 3.5. Serum Uric Acid Level Associated with Progression from Non-Severe to Severe COVID-19

Multivariable Cox proportional hazards regression model results are presented in Table 2. A significant association of low serum uric acid level at admission with a higher rate of severe COVID-19 progression was noted, which was independent of the observed potential confounders [HR for decrease of 1 mg/dL 1.279, 95% confidence interval (CI) 1.021–1.602; *p* = 0.032]. Furthermore, no remarkable inconsistency was observed after dividing the patients into subgroups based on sex and use of a uric acid-lowering agent (*p* values for interaction 0.948 and 0.473, respectively).

### 3.6. Serum Uric Acid Level Associated with Inflammation but Not Alveolar Damage or Coagulation Abnormality Markers

**Figure 1:**
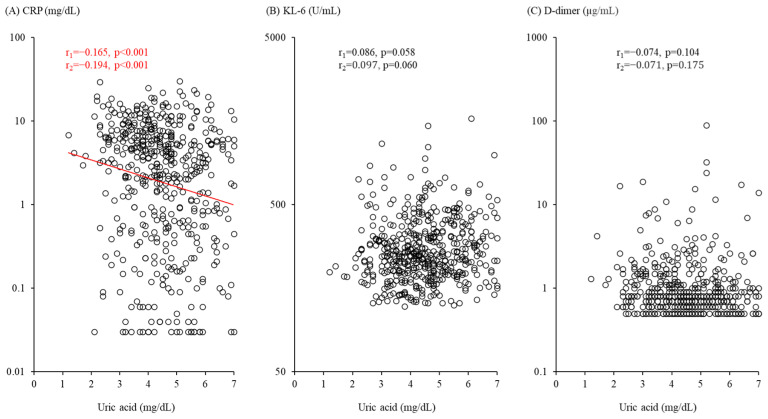
Associations of serum uric acid with level of (**A**) serum C-reactive protein (CRP), (**B**) serum Krebs von den Lungen-6 (KL-6), and (**C**) plasma D-dimer. r_1_ = Pearson’s correlation coefficient (standardized coefficient) from simple linear regression. r_2_ = Standardized coefficient from multiple linear regression, with the following variables included: age, sex, body mass index, smoking habit, presence of diabetes mellitus, hypertension, dyslipidemia, cerebrovascular and/or cardiovascular disease, chronic respiratory disease, estimated glomerular filtration rate, days from onset of illness to hospital admission, coronavirus disease 2019 severity at admission, and use of uric acid-lowering agents.

The values for serum uric acid level were plotted against markers of inflammation, alveolar damage, and coagulation abnormality (Figure 1). Simple regression analysis findings showed that serum uric acid level was inversely correlated with log CRP (r = −0.165, *p* < 0.001), whereas there was no significant correlation with log KL-6 (r = 0.086, *p* = 0.058) or log D-dimer (r = −0.074, *p* = 0.104) levels observed. Additionally, multiple linear regression analysis results showed that serum uric acid level was significantly associated with log CRP but not log KL-6 or log D-dimer levels, even after the application of adjustments for other variables (Figure 1).

### 3.7. Inflammation Shows Stronger Relationship with Progression from Non-Severe to Severe COVID-19 as Compared to Alveolar Damage and Coagulation Abnormality Markers

The associations of inflammation, alveolar damage, and coagulation abnormality markers with progression from non-severe to severe COVID-19 were examined using the same multivariable Cox proportional hazards regression model applied to serum uric acid level (Table 3). HR values were estimated based on a one standard deviation increase in each variable, then compared with each other. As compared with log KL-6 and log D-dimer, log CRP showed the strongest association with progression to severe COVID-19 (HR 2.079, 95% CI 1.389–3.113; *p* < 0.001).

### 3.8. Inflammation Marker Found to Influence Association of Serum Uric Acid Level with Severe COVID-19 Progression

The association of serum uric acid level at the time of admission with progression from non-severe to severe COVID-19 was re-evaluated with additional adjustments for the markers of inflammation, alveolar damage, and coagulation abnormality (Table 4). The results showed that the association of serum uric acid level with severe COVID-19 progression was significantly different from results obtained by analysis with and without inclusion of serum CRP level (HR = 1.337 vs. HR = 1.233; *p* = 0.041 by bootstrap method), whereas no statistically significant difference was noted for serum KL-6 or plasma D-dimer level, suggesting that inflammation influenced the association of uric acid with severe COVID-19 progression.

## 4. Discussion

In this study of patients initially hospitalized for non-severe COVID-19 and with a serum uric acid level ≤7 mg/dL at the time of admission, a low serum uric acid level was found to be significantly associated with a higher rate of progression to severe COVID-19, with no remarkable difference noted after stratification by sex or use of a uric acid-lowering agent. Furthermore, there was an inverse association of serum uric acid with serum CRP noted, but not with serum KL-6 or plasma D-dimer level. In addition, the association of serum uric acid level with severe COVID-19 progression was significantly different with and without inclusion of serum CRP, but not of serum KL-6 or plasma D-dimer level. These results suggest that low uric acid contributes to severe COVID-19 progression via increased inflammation in subjects without hyperuricemia.

Several previous studies have reported associations of low serum uric acid level with severity and mortality in COVID-19 patients [12,13,14], as well as the severity of other infectious diseases such as intra-abdominal sepsis and candidemia [31,32]. The Japan COVID-19 Task Force cohort found that a low uric acid level was associated with an increased risk of mechanical ventilation in 1523 patients with COVID-19 [12]. Additionally, in a study of 1854 hospitalized patients with COVID-19, Chen B and colleagues noted that a low uric acid level was associated with an increased risk of composite outcome, ICU admission, and mechanical ventilation [13], while Li and colleagues found an association of low uric acid with an increased risk of in-hospital death in a cohort of 540 severe or critical COVID-19 patients [14]. Consistent with those reports, the present results also indicated that a low level of serum uric acid was significantly associated with higher rate of progression from non-severe to severe COVID-19 in the examined patients. However, no known studies have comprehensively investigated the association between serum uric acid level and COVID-19 severity, including its association with inflammation, alveolar damage, and/or coagulation abnormality, which are known to underly the pathogenesis of severe COVID-19 cases [6,7]. Therefore, the role of low uric acid in severity of COVID-19 remains unclear. In the present study, serum uric acid level was found to be significantly associated with the level of serum CRP, but not that of serum KL-6 or plasma D-dimer. Furthermore, serum CRP, but not serum KL-6 or plasma D-dimer level, had an influence on the association between serum uric acid level and severe COVID-19 progression. These results provide a deeper understanding of the mechanism underlying the association of low uric acid and severe COVID-19 progression, and indicate the importance of inflammation in that association.

The antioxidant property of uric acid may be a key factor related to the importance of inflammation in the relationship of low serum uric acid level with severe COVID-19 progression. Accumulating evidence suggests that oxidative stress caused by overproduction of ROS and disruption of the antioxidant system plays a crucial role not only in the pathogenesis of SARS-CoV-2 infection, but also in the severity of COVID-19 [33,34]. Oxidative stress has been implicated to be involved in inflammation via signaling mechanisms, including redox-sensitive activation of transcription factors such as nuclear factor-kappa B in respiratory viral infections [35,36], and is also thought to be related to inflammation in COVID-19 cases [37,38]. Inflammation has a critically important role in the severity of COVID-19 progression [6,7], with elevated levels of inflammatory cytokines as well as CRP secreted under the influence of inflammatory cytokines, such as interleukin-6 and tumor necrosis factor-alpha, observed in patients with severe COVID-19 [39,40], suggesting the involvement of a cytokine storm in severe COVID-19 progression [41]. Consistent with those reports, a strong association of CRP level with severe COVID-19 progression was observed in the present cohort. On the other hand, the administration of steroids, an anti-inflammatory medication, has been shown to reduce the level of severity [7,38,42]. In addition, other reports have noted that the administration of antioxidants resulted in reductions in inflammation and severity in respiratory viral infection cases [43,44,45], with similar effects expected in patients affected by COVID-19 [5,34]. Additionally, uric acid has been shown to gain an antioxidant property by scavenging ROS, such as singlet oxygen, and peroxyl and hydroxyl radicals, and is also known to provide approximately half of the free radical-scavenging capacity in the human body [8,46]. Furthermore, it has been reported that administration of uric acid precursors resulted in a suppression of inflammatory mediators through ROS elimination in indomethacin-induced enteropathy cases [15]. The present results along with those in previous reports suggest that a low level of uric acid is not able to exert anti-inflammatory effects by scavenging ROS via its antioxidant property, resulting in severe COVID-19 progression in patients without hyperuricemia.

On the other hand, renal uric acid excretion is known to be increased in patients with COVID-19 [47], suggesting a link to inflammation, as also seen in severe acute respiratory syndrome patients [48]. Therefore, it cannot be ruled out that the relationship of serum uric acid level and inflammation with COVID-19 severity observed in the present study reflects increased renal uric acid excretion due to inflammation in affected patients. To provide better clarification of the role of uric acid in inflammation and severe COVID-19 progression, an interventional study that examines the association of uric acid administration with inflammation and severe progression in patients with non-severe COVID-19 and without hyperuricemia will be needed.

While ROS have been reported to be related to alveolar damage and coagulation abnormality [4,5], the present study did not find a significant association of serum uric acid level with serum KL-6 or plasma D-dimer level, and there was also no evidence of an effect of KL-6 or D-dimer on the relationship of serum uric acid level with severe COVID-19 progression. Those results indicate that a low level of uric acid contributes to severe COVID-19 progression, without affecting alveolar damage or coagulation abnormality. However, median serum KL-6 and plasma D-dimer values were not higher than the reference values. In addition, only the levels of serum KL-6, as a marker of alveolar damage, and plasma D-dimer, a marker of coagulation abnormality, were used in the present study. Thus, the relationship of uric acid with alveolar damage and coagulopathy requires further investigation. 

There are important limitations to this study that should be noted. First, serum uric acid level was measured at the time of admission, and that level prior to the onset of COVID-19 was not considered in the analyses. Second, since vaccinations against SARS-CoV-2 for the general public began in late May 2021 in Osaka Prefecture, patients who had received such vaccination were not included. It is unfortunate that we were not able to conduct a survey of SARS-CoV-2 strains that affected the individual cases examined, as that made it impossible to examine the effects of vaccination against SARS-CoV-2 or related strains on the results obtained in the present study. Third, it is also important to point out that high-sensitivity CRP testing was not performed. Fourth, though the diagnosis of COVID-19 in each case was made using a test approved by the Japanese Ministry of Health, Labour and Welfare for use at hospitals or clinics, it was not possible to investigate the diagnostic methods employed for each patient. Therefore, the association of serum uric acid level, inflammation, alveolar damage, and coagulation abnormality markers with COVID-19 severity, as well as diagnostic methods, could not be analyzed. Fifth, it was not possible to fully investigate occurrences of death or extrapulmonary manifestations, including involvement of the renal, urogenital, gastrointestinal, hepatic, endocrine, immune, and/or neurological systems [49,50,51]. Finally, this study was conducted using patients treated at a single center. However, based on instructions provided by Osaka Prefecture, Osaka City Juso Hospital had agreed to accept patients with non-severe COVID-19 from throughout Osaka Prefecture; thus, there was no apparent selection bias related to the results.

## 5. Conclusions

The present findings regarding patients initially placed in a hospital for the treatment of non-severe COVID-19 indicate a significant association of a low level of serum uric acid (≤7 mg/dL) at the time of admission with higher rate of progression to severe COVID-19. However, an inverse association of uric acid level with CRP level was noted, while the association between uric acid level and progression to severe COVID-19 was significantly different with and without its inclusion. Together, the present results indicate that a low level of uric acid contributes to severe COVID-19 progression via increased inflammation in individuals without hyperuricemia.

## Figures and Tables

**Table 1:** Clinical characteristics of patients with coronavirus disease 2019 (COVID-19) (*n* = 488).

Parameter	Value
Age (all subjects), years	76.0 (57.0–82.0)
Male, *n*	254 (52.0%)
Age (males), years	71.5 (55.0–80.0)
Females, *n*	234 (48.0%)
Age (females), years	79.0 (61.5–85.0)
BMI, kg/m^2^	23.1 (21.0–25.7)
Smoker, *n*	193 (39.5%)
eGFR, mL/min/1.73 m^2^	67.0 (54.0–84.0)
Coexisting diseases	
Diabetes mellitus, *n*	167 (34.2%)
Hypertension, *n*	270 (55.3%)
Dyslipidemia>, *n*	264 (54.1%)
Cerebrovascular/cardiovascular disease, *n*	91 (18.6%)
Chronic respiratory disease, *n*	64 (13.1%)
Days from onset of illness to hospital admission	5.0 (3.0–8.0%)
COVID-19 severity	
Mild, *n*	211 (43.2%)
Moderate I, *n*	167 (34.2%)
Moderate II, *n*	110 (22.5%)
Uric acid, mg/dL	4.4 (3.6–5.4)
Use of uric acid-lowering agents, *n*	54 (11.1%)
CRP, mg/dL	3.33 (0.58–6.77)
>KL-6, U/mL	252.0 (198.0–337.0)
D-dimer, µ>g/mL	0.8 (0.6–1.2)

Values are expressed as median (interquartile range) for continuous variables or number (percentage) for categorical variables. Abbreviations: BMI, body mass index; eGFR, estimated glomerular filtration rate; CRP, C-reactive protein; KL-6, Krebs von den Lungen-6.

**Table 2:** Multivariable Cox proportional analysis of factors associated with severe coronavirus disease 2019 (COVID-19) progression.

Variables	HR (95% CI)	*p* Value
Age (per 10-year increase)	1.342 (1.096–1.642)	0.004
Male (ref. female)	2.103 (1.232–3.589)	0.006
BMI (per 1 kg/m^2^ increase)	1.053 (0.995–1.115)	0.074
Smoker (ref. non-smoker)	1.324 (0.823–2.131)	0.247
Diabetes mellitus (ref. absence)	1.014 (0.644–1.595)	0.953
Hypertension (ref. absence)	1.144 (0.682–1.920)	0.611
Dyslipidemia (ref. absence)	0.936 (0.604–1.450)	0.768
Cerebrovascular/cardiovascular disease (ref. absence)	1.080 (0.660–1.768)	0.759
Chronic respiratory disease (ref. absence)	0.704 (0.363–1.365)	0.299
eGFR (per 10 mL/min/1.73 m^2^ increase)	0.934 (0.819–1.066)	0.311
Days from onset of illness to hospital admission (per 1 day increase)	0.994 (0.925–1.068)	0.876
Moderate I COVID-19 on admission (ref. mild)	2.055 (1.119–3.773)	0.020
Moderate II COVID-19 on admission (ref. mild)	5.264 (2.922–9.481)	<0.001
Use of uric acid-lowering agent (ref. absence)	1.067 (0.579–1.966)	0.835
Uric acid (per 1 mg/dL decrease)	1.279 (1.021–1.602)	0.032

Abbreviations: HR, hazard ratio; CI, confidence interval; BMI, body mass index; eGFR, estimated glomerular filtration rate.

**Table 3:** Association of markers of inflammation, alveolar damage, and coagulation abnormality at time of admission with progression from non-severe to severe coronavirus disease 2019 (COVID-19).

Variable of Interest	Direction	Standardized HR	95% CI	*p* Value
Uric acid	1 SD decrease	1.337	1.025–1.743	0.032
CRP	1 SD increase in log scale	2.079	1.389–3.113	<0.001
KL-6	1 SD increase in log scale	1.292	1.040–1.606	0.021
D-dimer	1 SD increase in log scale	1.183	0.966–1.448	0.104

Hazard ratio (HR) values were adjusted for age, sex, body mass index, smoking habit, presence of diabetes mellitus, hypertension, dyslipidemia, cerebrovascular/cardiovascular disease, chronic respiratory disease, days from onset of illness to hospital admission, estimated glomerular filtration rate, severity of COVID-19 on admission, and use of uric acid-lowering agent. Abbreviations: CRP, C-reactive protein; KL-6, Krebs von den Lungen-6; SD, standard deviation; CI, confidence interval.

**Table 4:** Association of serum uric acid level at time of admission with progression from non-severe to severe coronavirus disease 2019 (COVID-19), with additional adjustments for markers of inflammation, alveolar damage, and coagulation abnormality.

Variable Adjusted	Standardized Uric Acid HR	95% CI	*p* Value	*p* * Value
CRP	1.233	0.941–1.616	0.128	0.041
KL-6	1.337	1.029–1.736	0.029	0.991
D-dimer	1.320	1.013–1.719	0.039	0.393

Hazard ratio (HR) values for a 1 standard deviation decrease in serum uric acid level were adjusted for one of C-reactive protein (CRP), Krebs von den Lungen-6 (KL-6), or D-dimer, as well as age, sex, body mass index, smoking habit, presence of diabetes mellitus, hypertension, dyslipidemia, cerebrovascular/cardiovascular disease, chronic respiratory disease, days from onset of illness to hospital admission, estimated glomerular filtration rate, severity of COVID-19 on admission, and use of uric acid-lowering agent. *p* * value = *p* value for hypothesis test of null hypothesis that HR value for uric acid in analyses with or without adjustment for markers of inflammation, alveolar damage, and coagulation abnormality are equal. Abbreviations: CI, confidence interval.

## Data Availability

The data presented in this study are available on request from the corresponding author. The data are not publicly available due to Ethics Committee permission.
